# Acute myocardial infarction and syncope in an 18-year-old athlete with an abnormal origin of the left coronary artery: a case report

**DOI:** 10.4076/1757-1626-2-8142

**Published:** 2009-09-10

**Authors:** Ronan MG Berg, Bente Brendorp, Thomas Kristensen, Morten Helvind, Steffen Helqvist

**Affiliations:** 1 Department of Cardiology, University Hospital RigshospitaletDK-2100 CopenhagenDenmark; 2 Centre of Inflammation and Metabolism, Department of Infectious Diseases, University Hospital Rigshospitalet, section M7641DK-2100 CopenhagenDenmark; 3 Department of Radiology, University Hospital RigshospitaletDK-2100 CopenhagenDenmark; 4 Department of Cardiothoracic Surgery, University Hospital RigshospitaletDK-2100 CopenhagenDenmark

## Abstract

We report a case of acute myocardial infarction and syncope in an 18-year-old athlete during high-performance exercise. A coronary arteriography and an angiographic computed tomography scan subsequently revealed a left coronary arterial origin from the right aortic sinus along with an intramural course of the left main stem. The patient was successfully treated with surgical unroofing of the left main stem from inside the aorta. To our knowledge, this is the first report demonstrating this type of anomaly pre- and postoperatively by use of angiographic computed tomography scan in the context of acute coronary syndrome.

## Introduction

Coronary anomalies with a left coronary arterial origin from the right aortic sinus along with an intramural course of the left main stem have previously been described in autopsy materials from patients suffering a sudden cardiac death during high-performance exercise [1]. Here, we report a case of exercise-related acute myocardial infarction in which this type of anomaly was demonstrated by means of angiographic CT scanning.

## Case presentation

An 18-year-old Danish man of Ghanesian ancestry was admitted due to acute chest pain and a syncope occurring during vigorous exercise playing basket ball. The patient was unconscious for approximately one minute, and complained from sustained chest pain and dyspnea after regaining consciousness. There was no history of smoking or drug abuse. He had no previous medical history and no familial history of cardiovascular disease or sudden death; however, he had previously experienced episodes of chest pain and dyspnea without syncope during exercise.

Upon admission the patient had ongoing chest pain. Values for capillary oxygen saturation fluctuated between 82 and 94% with nasal oxygen supply. The arterial blood pressure was 70/40 mmHg, increasing to 125/75 mmHg after the administration of intravenous saline. There was no fever, and other vital signs were unremarkable. Chest examination demonstrated a regular heart rate (pulse 85) with no murmurs and normal breath sounds. The patient did not exhibit cyanosis or edema.

**Figure 1.**
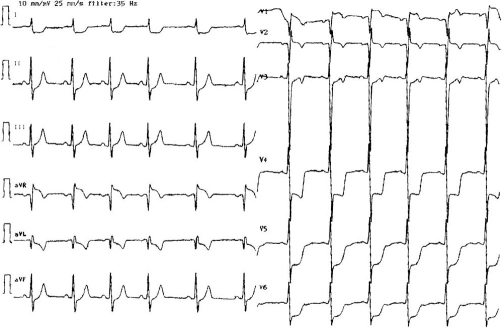
Initial 12-lead ECG obtained during chest pain upon admission.

The initial electrocardiogram (ECG) showed sinus rhythm with incomplete right bundle branch block, signs of left ventricular hypertrophy and severe myocardial ischemia with massive ST-segment depression in anterolateral leads along with ST-segment elevation in leads V_1_-V_2_ and aVR (Figure 1). In a subsequent ECG obtained after the chest pain had abated, the incomplete right bundle branch block and ST changes had ceased.

There were increases in coronary enzymes with a troponin T (TNT) of 0.25 μg/L (affirmative for myocardial infarction above 0.01 μg/L) and creatine kinase MB (CKMB) of 25 μg/L (affirmative for myocardial infarction above 5 μg/L). 12 hours later TNT and CKMB had increased further to 2.85 μg/L and 185 μg/L, and at 24 hours they had decreased to 1.80 μg/L and 68 μg/L, respectively. The leucocyte count was 15 × 10^9^ cells/L, and CRP was normal. There were no signs of thrombophilia or coagulopathy.

A chest radiograph showed a mild enlargement of the heart and a minor diffuse infiltrate near the apex of the left lung. Subsequent chest radiographs were normal. Bedside transthoracal echocardiography showed no abnormalities.

**Figure 2.**
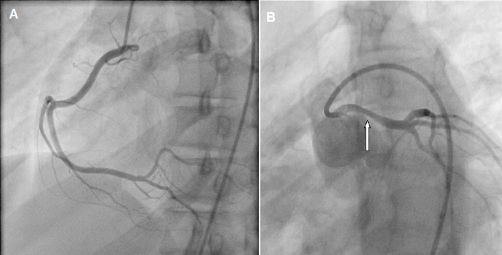
Coronary arteriography showing right side dominance and LM stenosis. **(A)** Right coronary artery. **(B)** Non selective catheterisation of the left coronary artery.

**Figure 3.**
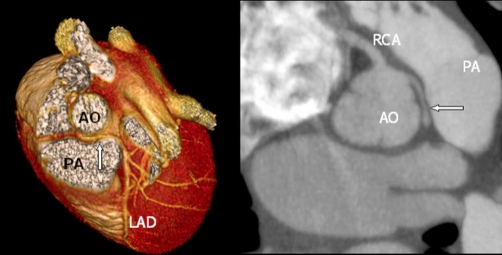
Preoperative ECG-gated 64-slice angiographic CT scan. Volume rendered image (left) and axial image (right) showing the origin and course of the LM (arrow) in relation to aorta (AO) and the pulmonary artery (PA). LAD: Left anterior descending. RCA: Right coronary artery.

A coronary arteriography (Figure 2) and an angiographic CT scan (Figure 3) were performed. The coronary arteriography raised the suspicion of an abnormal left coronary artery; this was confirmed by the angiographic CT scan which showed the origin of the left main stem (LM) from the right aortic sinus (Figure 2B and 3). The left coronary artery coursed aberrantly between the pulmonary trunk and aorta, hence causing a 30% LM stenosis (Figures 2B and 3, arrows). The initial ECG pattern during chest pain was consistent with LM stenosis [2]. However, a 30% LM stenosis is not sufficient to cause such profound ECG changes per se, which suggests that the LM was further narrowed during exercise.

**Figure 4.**
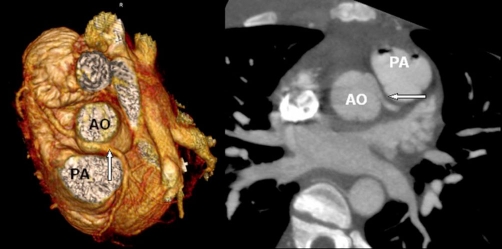
ECG-gated 64-slice angiographic CT scan performed after the operation demonstrates the unroofed left coronary artery (arrow) and confirms the patency. Volume rendered image (left) and axial image (right) showing the origin from the right aortic sinus and the proximal course of LM (arrow) between the aorta (AO) and the pulmonary artery (PA). LAD: Left anterior descending. RCA: Right coronary artery.

Open heart surgery revealed that the LM was located intramurally in the aortic tunica media, the internal orifice being in the right coronary sinus and the external orifice in the normal position of the left coronary artery. The patient was treated with surgical unroofing of the LM from inside the aorta, opening an orifice in the normal position of the LM in the left coronary sinus. Postoperatively, a CT angiography scan demonstrated the unroofed left coronary artery (Figure 4). The patient recovered completely and suffered no subsequent symptoms during six months of follow-up.

## Discussion

Myocardial ischemia is generally believed to be the underlying cause of sudden cardiac death in relation to coronary anomalies [1,3-5]. The mechanistic link between a coronary anomaly and myocardial ischemia has been a matter of dispute [3-5]; both kinking of the coronary artery, flap-like closure of the abnormal slit-like coronary lumen, and compression of the coronary artery between the aorta and the pulmonary trunk have been suggested [3-5]. In the present case, the LM was located intramurally in the aortic tunica media, and the aortic expansion during exercise would therefore expectedly aggravate the narrowing of the LM and, in synergy with an increased myocardial oxygen demand, lead to myocardial ischemia.

A left coronary arterial origin from the right aortic sinus, and a right coronary arterial origin from the left coronary sinus are the most common congenital coronary malformations that have been described in autopsy materials from previously healthy athletes who suffered a sudden death [1,6]. Furthermore, an intramural course of the left coronary artery in the aorta has been noted in several of the cases [1], and has previously been demonstrated in the context of exercise-related acute myocardial infarction [7]. These malformations are associated with sudden cardiac death to a wider extent in competitive athletes than in non-athletes [6]. This lends further support to the hypothesis that compression of the coronary artery during workout is a common pathogenetic link in exercise related death in these patients. In this context, the present case is unique, as it demonstrates this concept in the clinical setting of acute coronary syndrome with myocardial infarction by means of angiographic CT scanning.

The incidence of athletic field deaths ranges between 0.5 and 1.6 out of 100.000 young competitive athletes per year [6,8]. The underlying diseases are mainly hypertrophic cardiomyopathy, arrythmogenic right ventricular dysplasia and congenital coronary malformations [6,8]. As the latter is amenable to surgical correction, it is crucial to be aware of this cause of exercise-induced chest pain, dyspnea and syncope in healthy young subjects.
